# Combat exposure and behavioral health in U.S. Army Special Forces

**DOI:** 10.1371/journal.pone.0270515

**Published:** 2022-06-28

**Authors:** Anna C. Rivera, Cynthia A. LeardMann, Rudolph P. Rull, Adam Cooper, Steve Warner, Dennis Faix, Edwin Deagle, Rob Neff, Ryan Caserta, Amy B. Adler, for the Millennium Cohort Study Team

**Affiliations:** 1 Deployment Health Research Department, Naval Health Research Center, San Diego, California, United States of America; 2 Leidos, San Diego, California, United States of America; 3 Innovative Employee Solutions, San Diego, California, United States of America; 4 Naval Health Research Center, San Diego, California, United States of America; 5 Preservation of the Force and Family, United States Special Operation Command Headquarters, MacDill Air Force Base, Tampa, Florida, United States of America; 6 Walter Reed Army Institute of Research, Silver Spring, Maryland, United States of America; Behavioral Biology Center for Military Psychiatry and Neuroscience, UNITED STATES

## Abstract

Although combat has been found to be associated with adverse health outcomes, little is known about the impact of specific combat exposures, particularly among specialized personnel. This study examined the association of different types of combat exposures with behavioral health outcomes, and whether these associations differed by Army occupational specialization: General Purpose Forces infantrymen (n = 5,361), Ranger Qualified infantrymen (n = 308), and Special Forces personnel (n = 593). Multivariable regression models estimated the association of combat severity, type of combat event (fighting, killing, threat to oneself, death/injury of others), and type of killing with mental health disorders, trouble sleeping, and problem drinking. Combat severity, each type of combat event, and killing noncombatants were associated with adverse health outcomes after adjusting for covariates and other combat exposures. Except for trouble sleeping, these associations did not differ by occupational specialization, though the prevalence and odds of outcomes were generally lower for Special Forces personnel.

## Introduction

Service members who deploy to active conflicts often experience combat, which has been consistently associated with subsequent adverse health outcomes, including mental health problems [1–5], sleep problems [6, 7], and alcohol misuse [8, 9]. High combat severity, measured using the number of combat experiences endorsed, has been associated with posttraumatic stress disorder (PTSD) [10] and alcohol-related problems [10, 11]. While the majority of these previous studies have broadly defined combat exposure, the investigation of more specific exposures is warranted in order to inform appropriate training, early intervention, and treatment.

When combat exposure is examined in terms of specific event types, instead of broadly as described above, evidence suggests that certain exposures may drive associations between combat and adverse health outcomes. Specific combat events, such as fighting, killing, threat to oneself, and death/injury to others, have been associated with particular patterns of mental health outcomes, such as PTSD [10, 12–16], suicide [17], and alcohol misuse [18, 19]. One health outcome that has not been assessed in relation to types of combat events is sleep problems, despite evidence for its association with general combat exposure [6, 7]. It is important to assess sleep in military populations given the abundance of operational and environmental sleep disturbances (e.g., training for long hours, harsh sleeping surfaces) [20] and the effect of poor sleep on subsequent health problems, as well as military readiness and performance [21, 22].

Among the specific types of combat events, one of the most complex is killing. The experience of killing in combat has been associated with PTSD [16], suicidal behaviors [17, 23–25], and alcohol misuse [18, 19, 26]. However, most of these previous studies combined different types of killing into a single measure, despite research suggesting that killing a noncombatant may be associated with a greater magnitude of mental health problems compared with killing an enemy combatant [10, 25, 27–29]. Few studies have examined this distinction, although identifying unique risk factors can inform intervention strategies.

Another limitation of the existing literature is that studies linking combat exposure and behavioral health mostly focused on conventional forces. While conventional forces represent the majority of military personnel in combat, personnel in the Special Operations Forces, such as those in the Army Special Forces (SF), are essential to enacting military strategy by undertaking sensitive missions with high stakes. These specialized units are assigned to high-priority, hostile, and politically sensitive missions and are the primary option for operations that include elements of time sensitivity, regional expertise, clandestinity, and/or high degrees of risk [30–33]. Due to the nature of Special Operation Forces, SF personnel are carefully selected and trained; they must pass an intense selection process and complete the 12–18 months Special Forces Qualification Course [34]. Given all these factors, SF have a unique culture that emphasizes mission, cohesion, and mental toughness. Even though SF personnel are expected to perform in high-risk operational contexts and may be exposed to extreme levels of stress, previous research has found SF personnel report fewer adverse mental and physical health outcomes compared with other soldiers [34–36]. While it is possible that some of these differences may be related to demographic or sociocultural characteristics, one recent prospective study observed that even after adjusting for baseline demographic, military, deployment, mental health, and physical health factors, SF personnel were significantly healthier compared with infantrymen serving in conventional units [34].

Comparisons with other military personnel who volunteer for, are selected into, and complete intense and arduous training courses are potentially informative. One example of such personnel is Ranger Qualified (RQ) infantrymen who complete a 9-week premier leadership course (i.e., Army Ranger School) but continue to serve with conventional units and thus are not considered members of the Special Operations Forces. Despite high exposure to combat, these RQ infantrymen also report fewer mental and physical health problems compared with soldiers who complete standard training and serve in conventional units, such as General Purpose Forces (GPF) infantrymen [34]. In order to appropriately tailor intervention strategies, it is important to understand if the impact of combat differs by occupational specialization.

Building on the evidence documenting the link between specific categories of combat exposure and behavioral health outcomes, the present study was designed to (1) expand the scope of outcomes to include sleep problems, (2) examine the impact of type of killing on behavioral health outcomes, and (3) determine if these associations differ by occupational specialization. Leveraging data from a large military cohort, the first aim was to investigate the association of combat exposures (combat severity, types of combat events, and type of killing) with mental health disorders, trouble sleeping, and problem drinking among soldiers serving in combat roles. The second aim was to investigate if these associations between types of combat exposures and behavioral health outcomes differed by type of occupational specialization (GPF infantrymen, RQ infantrymen, or SF personnel). Results from this study can be used to inform targeted screening, prevention, and intervention efforts.

## Materials and methods

### Study population

The Millennium Cohort Study, the largest prospective cohort study of military personnel and veterans, was designed to investigate the effects of military service on health over time [37–39]. Since its launch in 2001, the study has enrolled participants from all service branches, including active duty, Reserve, and National Guard personnel. Over 200,000 participants were enrolled in the first four panels between 2001 and 2013 (panel 1, July 2001-June 2003; panel 2, June 2004-February 2006; panel 3, June 2007-December 2008; panel 4, June 2011-April 2013; 27.3% cumulative baseline response rate). Previous studies have found the Millennium Cohort to be a representative sample of service members in terms of health status, and analyses on weighting for nonresponse have not identified changes in metrics for mental disorders [40, 41]. Participants were asked to complete a baseline survey at enrollment and a follow-up survey approximately every 3 to 5 years covering a broad scope of topics, including military experiences, lifestyle characteristics, and physical, mental, and behavioral health outcomes. Surveys are linked to demographic, occupation, and deployment data from the Defense Manpower Data Center. A more detailed description of the study methodology has been provided elsewhere [37–39]. The study protocol was approved by the Naval Health Research Center Institutional Review Board in compliance with all applicable Federal regulations governing the protection of human subjects (NHRC.2000.0007). All participants provided written voluntary informed consent.

The sample for this study was restricted to U.S. Army personnel in one of three occupational specializations (GPF infantrymen, RQ infantrymen, or SF personnel) who had completed at least one deployment in support of the conflicts in Iraq or Afghanistan (n = 7,104). This study used definitions for these three Army occupational specializations that have been previously described [34]. In summary, SF personnel included Weapons, Engineer, Communications, Intelligence, and Medical Sergeants who passed a selection course and completed a 12–18 months Special Forces Qualification Course; RQ infantrymen included those who had completed Ranger School and were awarded the Ranger Tab; and GPF infantrymen included only non-RQ infantrymen. Soldiers who did not complete at least one survey following attainment of one of the three occupational specializations (n = 422), were women (n = 32), or separated from military service before an occupational specialization was attained (n = 174) were excluded. Of the 6,476 remaining eligible participants, those who did not respond to the surveys with the combat questions (n = 96) or were missing all combat items (n = 34) were also excluded. Lastly, to ensure homogeneous comparison groups, soldiers were excluded from all analyses if they had a Unit Identification Code identifying them as members of the 75th Ranger Regiment (n = 84) because whereas these soldiers are Ranger qualified, they are Special Operations Forces based on their unit affiliation. Conversely, RQ and GPF infantrymen serve along conventional forces, while SF personnel serve in specialized units. The final study population consisted of 6,262 participants (96.7% of the eligible sample), including 5,361 GPF infantrymen, 308 RQ infantrymen, and 593 SF personnel. For each analysis, those missing the outcome or exposure of interest were removed, resulting in a slightly different analytic population for each model.

### Measures

Mental health disorders, trouble sleeping, and problem drinking were assessed using data from Millennium Cohort surveys, using the same approach as a previous study [34]. Probable mental health disorder was an aggregate outcome based on four disorders: probable PTSD, depression, panic, or anxiety. Probable PTSD was based on the 17-item PTSD Checklist–Civilian Version, using Diagnostic and Statistical Manual of Mental Disorders, Fourth Edition (DSM-IV) criteria [34, 42, 43]. Probable PTSD was defined as those who reported “moderately” or higher on at least one intrusion item, two hyperarousal items, and three avoidance items. Scored using the standardized scoring algorithms, probable depression, panic, and anxiety were assessed using three separate subscales from the Patient Health Questionnaire (PHQ) [34, 44]. Depression was defined as those who endorsed five or more of the eight PHQ items as “more than half the days” or “nearly every day”, including the endorsement of anhedonia or depressed mood in the last two weeks [45]. Probable panic syndrome was defined as those who answered “yes” (versus “no”) to the first four PHQ items (e.g., In the last 4 weeks, have you had an anxiety attack–suddenly feeling fear or panic) as well as at least four of the 11 anxiety attack symptoms (e.g., were you short of breath) in the last four weeks [44, 46]. Probable anxiety was defined as those who endorsed “more than half the days” to “feeling nervous, anxious, on edge, or worrying a lot about different things" and three or more of the other six anxiety symptoms (e.g., Feeling restless so that it is hard to sit still). Trouble sleeping was defined as those who endorsed trouble falling asleep or staying asleep over the last 4 weeks [21]. Problem drinking was defined as those who endorsed any of the five PHQ alcohol items (e.g., drove a car after having several drinks or after drinking too much) more than once in the last 12 months [44].

Combat exposure was analyzed in three contexts: (1) combat severity, (2) four types of combat events (fighting, killing, threat to oneself, and death/injury of others), and (3) type of killing (enemy combatant, noncombatant). These exposures were derived from 12 combat experience items on the survey (e.g., being attacked or ambushed, handling human remains), which are a core subset of the items assessed by the Walter Reed Army Institute Research Land Combat Study questionnaire [1, 47]. Combat severity was defined as the number of individual combat items endorsed. Due to small population sizes, combat severity was dichotomized as low combat severity (0–6 combat items endorsed) and high combat severity (7–12 items endorsed). Type of combat event was assessed as four separate binary variables: fighting, killing, threat to oneself, and death/injury of others, as defined previously [18, 48]. Each type of combat event comprised a subset of the 12 combat items. Fighting consisted of three items (being attacked or ambushed, receiving small arms fire, or clearing/searching buildings). Killing consisted of being directly responsible for the death of an enemy combatant or being directly responsible for the death of a noncombatant. Threat to oneself consisted of having an improvised explosive device explode near you or being wounded or injured. Death/injury consisted of five items (seeing dead bodies, handling human remains, knowing someone injured/killed, seeing Americans injured/killed, or having a unit member injured/killed). For each type of combat event, participants who self-reported one or more items were classified as experiencing that type of combat event. The final analysis focused on type of killing because previous research has found differences between those who have been directly responsible for the death of a noncombatant compared with an enemy combatant [10, 25, 27, 28]. Type of killing was categorized as (1) “neither,” (2) “enemy combatant only,” or (3) “noncombatant.” Most of those who identified as being responsible for the death of a noncombatant were also responsible for the death of an enemy combatant (94%). To facilitate comparison between those who had killed a noncombatant versus an enemy combatant, the reference category for these analyses was “enemy combatant only.”

Based on the cross-sectional design of the study and availability of the 12 combat items on surveys, one survey from either the 2007–2008 or 2011–2013 survey cycle was used as the assessment point for all analyses. These survey cycles were a baseline/enrollment survey or a follow-up survey, depending on the panel. The earlier survey was used as the assessment point unless one of the following exceptions was met, resulting in the second survey being used for assessment: the participant (1) was not in the specific occupational specialization (i.e., GPF infantrymen, RQ infantrymen, SF personnel) at time of earlier survey, (2) was missing combat data on the earlier survey (a non-responder or skipped combat items), or (3) did not endorse any combat items on the earlier survey but did endorse at least one combat item on the second survey. All variables (exposures, outcomes, and covariates) were measured at the assessment point.

Covariates included demographics (race and ethnicity, marital status, education, age, and panel) and military service characteristics (service component and rank). Demographics and military characteristics were obtained from Department of Defense personnel records obtained from Defense Manpower Data Center, with the exception of marital status and education, which were obtained from the Millennium Cohort Study survey.

### Statistical analyses

Descriptive analyses were conducted to compare demographic and military characteristics, combat severity, type of combat event, type of killing, and outcomes by occupational specialization (i.e., GPF infantrymen, RQ infantrymen, SF personnel). Adjusted odds ratios (AORs) and 95% confidence intervals (CIs) were estimated using multivariable logistic regression. Covariates (i.e., occupational specialization, race and ethnicity, marital status, education, age, panel, component, and rank) were selected a priori and remained in all multivariable models regardless of statistical significance to facilitate comparisons between groups. First, the association between occupational specialization and each outcome was investigated, adjusting for covariates. Next, the association of each combat exposure with each of the three outcomes was examined, using three types of models. Specifically, combat severity was the exposure of interest in Model 1. In Model 2, all types of combat events (fighting, killing, threat to oneself, death/injury of others) were examined and mutually adjusted in a single model. In Model 3, type of killing (neither, enemy combatant only, noncombatant) was the exposure of interest, with adjustment for fighting, threat to oneself, and death/injury of others.

To determine if the association between the exposure of interest and the outcomes were moderated by occupational specialization, an interaction term was included in each model (occupational specialization*combat exposure). If the interaction term was significant (*p* < 0.05), the model was stratified by occupational specialization. A false discovery rate adjustment method was applied to account for multiple comparisons. When conducting multiple comparisons, false discovery rate adjustment procedures control the expected proportions of type I errors in null hypothesis testing, which are slightly less stringent than Bonferroni corrections but have greater power [49].

The variance inflation factor was used to assess for collinearity in all models, where a value greater than 4 indicated possible collinearity. All four enrollment panels were pooled for these analyses. All data analyses and manipulation were performed using SAS software version 9.4 (SAS Institute Inc., Cary, NC).

## Results

Descriptive characteristics by occupational specialization are shown in Table 1. Across all occupational specializations, most participants were non-Hispanic White, married, active duty, enlisted rank, endorsed 7–12 combat items, and endorsed fighting, threat to oneself, and death/injury of others. Most participants did not endorse killing and correspondingly were in the “neither” type of killing category. In general, the level and type of combat endorsed was fairly consistent across occupational specializations, given that soldiers in all three occupational specializations examined serve in combat roles. RQ infantrymen and SF personnel were slightly more educated and older than GPF infantrymen. GPF infantrymen were more likely to be unmarried, Reservists, and enlisted rank compared with RQ infantrymen and SF personnel. GPF infantrymen had the highest proportion of mental health disorders compared with RQ infantrymen, followed by SF personnel (22.9% vs. 8.1% vs. 6.6%, respectively). A similar trend was observed for the trouble sleeping and problem drinking outcomes (Table 1).

**Table 1:** Characteristics of General Purpose Forces infantrymen, Ranger Qualified infantrymen, and Special Forces personnel.

Characteristic	General Purpose Forces	Ranger Qualified infantrymen	Special Forces personnel
	n = 5,361	n = 308	n = 593
	n (%)	n (%)	n (%)
Demographics			
Race and ethnicity*			
Hispanic	373 (7.0)	18 (5.8)	22 (3.7)
White, non-Hispanic	4,434 (82.7)	255 (82.8)	528 (89.0)
Other^a^	554 (10.3)	35 (11.4)	43 (7.3)
Marital status*			
Not married	2,017 (37.6)	82 (26.6)	163 (27.5)
Married	3,344 (62.4)	226 (73.4)	430 (72.5)
Education*			
High school degree or less	1,453 (27.1)	28 (9.1)	37 (6.2)
Some college/associate	2,484 (46.3)	105 (34.1)	233 (39.3)
Bachelor’s or higher	1,424 (26.6)	175 (56.8)	323 (54.5)
Age*, mean, SD	30.2, 7.9	32.6, 8.0	34.2, 8.9
Panel*^,^^b^			
1	1,479 (27.6)	148 (48.1)	290 (48.9)
2	711 (13.3)	55 (17.9)	64 (10.8)
3	1,153 (21.5)	36 (11.7)	95 (16.0)
4	2,018 (37.6)	69 (22.4)	144 (24.3)
Military service			
Component*			
Active duty	3,212 (59.9)	260 (84.4)	478 (80.6)
Reserves	2,149 (40.1)	48 (15.6)	115 (19.4)
Rank*			
Enlisted	4,385 (81.8)	159 (51.6)	374 (63.1)
Officer	976 (18.2)	149 (48.4)	219 (36.9)
Combat severity*^,^^c^ (No. of combat items endorsed)			
Low (0–6)	2,119 (39.5)	101 (32.8)	251 (42.3)
High (7–12)	3,242 (60.5)	207 (67.2)	342 (57.7)
Type of combat event			
Fighting^d^			
No	769 (14.4)	35 (11.4)	88 (14.8)
Yes	4,587 (85.6)	273 (88.6)	505 (85.2)
Killing^e^			
No	3,179 (59.7)	173 (56.2)	361 (61.3)
Yes	2,149 (40.3)	135 (43.8)	228 (38.7)
Threat to oneself*^,^^f^			
No	1,767 (33.0)	107 (34.7)	277 (46.8)
Yes	3,586 (67.0)	201 (65.3)	315 (53.2)
Death/injury of others^g^			
No	694 (13.0)	30 (9.7)	62 (10.5)
Yes	4,660 (87.0)	278 (90.3)	531 (89.5)
Type of killing*^,^^h^			
Neither	3,179 (59.7)	173 (56.2)	361 (61.3)
Enemy combatant only	1,672 (31.4)	103 (33.4)	199 (33.8)
Noncombatant	477 (9.0)	32 (10.4)	29 (4.9)
Outcomes			
Mental health disorders*^,^^i^			
No	4,126 (77.1)	280 (91.8)	553 (93.4)
Yes	1,227 (22.9)	25 (8.2)	39 (6.6)
Trouble sleeping*^,^^j^			
No	2,728 (51.0)	219 (71.6)	445 (75.3)
Yes	2,622 (49.0)	87 (28.4)	146 (24.7)
Problem drinking*^,^^k^			
No	4,232 (78.3)	266 (86.6)	539 (91.4)
Yes	1,107 (20.7)	41 (13.4)	51 (8.6)

IED, improvised explosive device; PHQ, Patient Health Questionnaire; PTSD, posttraumatic stress disorder.

All covariates were measured at the assessment point.

Note: Column totals may not sum to population total due to exclusions of missing values. Due to nonresponse, the number of missing responses on each outcome variable vary. Sample sizes were as follows: mental health disorders n = 6,250, trouble sleeping n = 6,247, problem drinking n = 6,236.

* *p* < .05 for chi-square test of independence between each characteristic and occupational specialization.

^a^ “Other” included 64 participants (1.0%) identifying as American Indian, 248 (4.0%) as Asian or Pacific Islander, 280 (4.5%) as Black, non-Hispanic, and 40 (0.7%) as non-Hispanic multiracial.

^b^ This study used four panels of Millennium Cohort Study participants.

^c^ Combat severity is the categorized sum of the number of combat items endorsed from the following list: being attacked or ambushed, receiving small arms fire, clearing/searching buildings, killed an enemy combatant, killed a noncombatant, having an IED explode near you, being wounded or injured, seeing dead bodies, handling human remains, knowing someone injured/killed, seeing Americans injured/killed, or having a unit member injured/killed.

^d^ Fighting includes being attacked or ambushed, receiving small arms fire, or clearing/searching buildings.

^e^ Killing includes being directly responsible for the death of an enemy combatant or a noncombatant.

^f^ Threat to oneself includes having an IED explode near you or being wounded or injured.

^g^ Death/injury of others includes seeing dead bodies, handling human remains, knowing someone injured/killed, seeing Americans injured/killed, or having a unit member injured/killed.

^h^ Type of killing was categorized as “neither,” “enemy combatant only,” and “noncombatant.” Almost all who reported being responsible for the death of a noncombatant also reported being responsible for the death of an enemy combatant.

^i^ Mental health disorders is defined as endorsement of PTSD, depression, panic, or anxiety.

^j^ Trouble sleeping is based on an endorsement of having trouble falling asleep or staying asleep.

^k^ Problem drinking is based on endorsement of any of the five PHQ alcohol items.

SF personnel were significantly less likely to have mental health disorders, trouble sleeping, and problem drinking than GPF infantrymen after adjusting for covariates. RQ infantrymen were similarly less likely to have mental health disorders and trouble sleeping than GPF infantrymen (Table 2). Adjusted odds ratios (AORs) of mental health disorders, trouble sleeping, and problem drinking for each type of combat exposure are shown in Table 3. Model 1 indicated that those who experienced high combat severity were more likely to experience mental health disorders (AOR = 2.90, 95% CI [2.48, 3.38]), trouble sleeping (AOR = 2.13, 95% CI [1.90, 2.38]), and problem drinking (AOR = 1.43, 95% CI [1.23, 1.65]) compared with those who experienced low combat severity (Table 3).

**Table 2:** Adjusted odds ratios for screening positive for mental health disorders, trouble sleeping, and problem drinking by occupational specialization.

	Mental health disorders^a^	Trouble sleeping^b^	Problem drinking^c^
	AOR (95% CI)	AOR (95% CI)	AOR (95% CI)
Occupational specialization^d^	n = 6,250	n = 6,247	n = 6,236
GPF infantrymen	1.00	1.00	1.00
RQ infantrymen	**0.47 (0.31, 0.72)**	**0.55 (0.42, 0.71)**	0.82 (0.58, 1.17)
SF personnel	**0.33 (0.23, 0.46)**	**0.41 (0.33, 0.50)**	**0.49 (0.36, 0.66)**

^a^ Mental health disorders is defined as endorsement of PTSD, depression, panic, or anxiety.

^b^ Trouble sleeping is based on an endorsement of having trouble falling asleep or staying asleep.

^c^ Problem drinking is based on endorsement of any of the five PHQ alcohol items.

^d^ A separate model was run for each outcome. Each model was adjusted for race and ethnicity, marital status, education, age, panel, component, and rank.

**Table 3:** Adjusted odds ratios for screening positive for mental health disorders, trouble sleeping, and problem drinking by type of combat exposure.

Model	Mental health disorders^a^	Trouble sleeping^b^	Problem drinking^c^
	AOR (95% CI)	AOR (95% CI)	AOR (95% CI)
Model 1: Combat severity^d^	n = 6,250	n = 6,247	n = 6,236
Low (0–6 items)	1.00	1.00	1.00
High (7–12 items)	**2.90 (2.48, 3.38)**	**2.13 (1.90, 2.38)**	**1.43 (1.23, 1.65)**
Model 2: Type of combat event^e^	n = 6,204	n = 6,201	n = 6,190
Fighting^f^			
No	1.00	1.00	1.00
Yes	0.97 (0.72, 1.29)	1.10 (0.91, 1.34)	**1.53 (1.17, 2.00)**
Killing^g^			
No	1.00	1.00	1.00
Yes	**2.14 (1.85, 2.48)**	**1.83 (1.62, 2.07)**	**1.27 (1.10, 1.47)**
Threat to oneself^h^			
No	1.00	1.00	1.00
Yes	**1.51 (1.25, 1.82)**	**1.29 (1.13, 1.48)**	0.93 (0.79, 1.11)
Death/injury of others^i^			
No	1.00	1.00	1.00
Yes	**1.82 (1.32, 2.49)**	**1.35 (1.10, 1.64)**	1.27 (0.97, 1.66)
Model 3: Type of killing^j^^,^^k^	n = 6,204	n = 6,201	n = 6,190
Neither	**0.56 (0.48, 0.65)**	**0.61 (0.54, 0.69)**	0.95 (0.81, 1.11)
Enemy combatant only	1.00	1.00	1.00
Noncombatant	**2.14 (1.74, 2.63)**	**1.75 (1.42, 2.16)**	**2.12 (1.71, 2.63)**

AOR, adjusted odds ratio; CI, confidence interval; IED, improvised explosive device; PHQ, Patient Health Questionnaire; PTSD, posttraumatic stress disorder.

Significant results are shown in bold.

^a^ Mental health disorders is defined as endorsement of PTSD, depression, panic, or anxiety.

^b^ Trouble sleeping is based on an endorsement of having trouble falling asleep or staying asleep.

^c^ Problem drinking is based on endorsement of any of the five PHQ alcohol items.

^d^ Model 1 was adjusted for occupational specialization, race and ethnicity, marital status, education, age, panel, component, and rank. A separate model was run for each outcome.

^e^ Model 2 was adjusted for occupational specialization, race and ethnicity, marital status, education, age, panel, component and rank. A separate model was run for each outcome.

^f^ Fighting includes being attacked or ambushed, receiving small arms fire, or clearing/searching buildings.

^g^ Killing includes being directly responsible for the death of an enemy combatant or a noncombatant.

^h^ Threat to oneself includes having an IED explode near you or being wounded or injured.

^i^ Death/injury of others includes seeing dead bodies, handling human remains, knowing someone injured/killed, seeing Americans injured/killed, or having a unit member injured/killed).

^j^ Type of killing was categorized as “neither,” “enemy combatant only,” and “noncombatant.” Almost all who reported being responsible for the death of a noncombatant also reported being responsible for the death of an enemy combatant.

^k^ Model 3 was adjusted for occupational specialization, fighting, threat to oneself, death/injury of others, race and ethnicity, marital status, education, age, panel, component and rank. A separate model was run for each outcome.

Results from Model 2 indicated those who experienced killing (enemy combatants and/or noncombatants) had significantly higher odds of all three outcomes compared with those who did not experience killing (AOR 1.27–2.14), after adjusting for fighting, threat to oneself, and death/injury of others. Those who experienced fighting were significantly more likely to report problem drinking, while those who experienced the threat to oneself and death/injury of others were significantly more likely to report both mental health disorders and trouble sleeping compared with those who did not experience these combat events, after adjusting for other types of combat events.

Results from Model 3 indicated those who were directly responsible for the death of a noncombatant were significantly more likely to experience all three outcomes compared with those who reported being responsible for the death of an enemy combatant only (AOR 1.77–2.19), after adjusting for fighting, threat to oneself, and death/injury of others (Table 3). Compared with those who were responsible for the death of an enemy combatant only, soldiers who reported no killing had lower odds of mental health disorders (AOR = 0.56, 95% CI [0.48, 0.65]) and trouble sleeping (AOR = 0.61, 95% CI [0.54, 0.69]).

In general, SF personnel and RQ infantrymen had lower odds of behavioral health outcomes compared with GPF infantrymen; however, the associations between type of combat exposures and outcomes were largely consistent across the different occupational specializations. Specifically, none of the interaction terms between type of combat exposure and occupational specialization were significant for mental health disorders and problem drinking (all *p* values >0.10; results not shown in tables) and thus stratified analyses were not conducted for these outcomes. However, interaction terms between three combat variables (combat severity, threat to oneself, and type of killing) and trouble sleeping were significant (*p* values = .001, .017, and .005, respectively), indicating that the association between certain combat exposures and trouble sleeping varied by occupational specialization. Therefore, subsequent analyses were stratified by occupational specialization for these three types of combat exposures with trouble sleeping (Table 4) and consequently, the corresponding non-stratified effect estimates in Table 3 should be interpreted with caution. In stratified analyses, GPF infantrymen who experienced high combat severity, threat to oneself, and being directly responsible for the death of a noncombatant were more likely to experience trouble sleeping compared with GPF infantrymen who did not have these combat exposures (Table 4). Similarly, SF personnel who experienced high combat severity and were directly responsible for the death of a noncombatant were more likely to experience trouble sleeping compared with SF personnel who did not endorse these combat exposures (Table 4). Interestingly, RQ infantrymen who endorsed these combat exposures did not appear to differ from RQ infantrymen who did not endorse these experiences with respect to trouble sleeping (Table 4). However, this may be driven by a smaller sample size and therefore less statistical power to detect a significant difference.

**Table 4:** Adjusted odds ratios for trouble sleeping stratified by occupational specialization.

Model	General Purpose Forces	Ranger Qualified infantrymen	Special Forces personnel
	n = 5,309	n = 306	n = 586
	AOR (95% CI)	AOR (95% CI)	AOR (95% CI)
Model 1: Combat severity^a^			
Low (0–6 items)	1.00	1.00	1.00
High (7–12 items)	**2.26 (2.00, 2.55)**	0.98 (0.54, 1.77)	**1.57 (1.05, 2.35)**
Model 2: Type of combat event^b^			
Threat to oneself^c^			
No	1.00	1.00	1.00
Yes	**1.38 (1.19, 1.60)**	0.64 (0.33, 1.26)	0.99 (0.63, 1.54)
Model 3: Type of killing^d^^,^^e^			
Neither	**0.61 (0.53, 0.70)**	0.81 (0.43, 1.55)	**0.46 (0.29, 0.73)**
Enemy combatant only	1.00	1.00	1.00
Noncombatant	**1.76 (1.41, 2.21)**	0.78 (0.29, 2.08)	**3.21 (1.40, 7.38)**

AOR, adjusted odds ratio; CI, confidence interval; IED, improvised explosive device.

Significant results are shown in bold.

^a^ Model 1 was adjusted for race and ethnicity, marital status, education, age, panel, component, and rank. A separate model was run for each occupational specialization.

^b^ Model 2 was adjusted for the other types of combat events (fighting, killing, and death/injury of others), race and ethnicity, marital status, education, age, panel, component and rank. A separate model was run for each occupational specialization.

^c^ Threat to oneself includes having an IED explode near you or being wounded or injured.

^d^ Type of killing was categorized as “neither,” “enemy combatant only,” and “noncombatant.” Almost all who reported being responsible for the death of a noncombatant also reported being responsible for the death of an enemy combatant.

^e^ Model 3 was adjusted for fighting, threat to oneself, death/injury of others, race and ethnicity, marital status, education, age, panel, component, and rank. A separate model was run for each occupational specialization.

A sub-analysis was conducted to examine the association between type of combat exposure and trouble sleeping among those who had completed a previous Millennium Cohort survey to adjust for a history of sleep apnea, as well as previous physical and mental health status. Overall, the effect estimates of the sub-analysis were similar to those of the main models (Table A in S1 Text). However, the association between each type of combat exposure and trouble sleeping was slightly attenuated and confidence intervals widened. Models could not be stratified by occupational specialization due to small sample sizes.

## Discussion

The present study provides important insights into the relationship between specific combat exposures and the risk of mental health disorders, trouble sleeping, and problem drinking among soldiers serving in various combat roles. While SF personnel and RQ infantrymen were less likely to have each outcome of interest compared with GPF infantrymen, findings indicated that high combat severity was associated with mental health disorders, trouble sleeping, and problem drinking. Similarly, some specific combat exposures such as killing—noncombatants in particular—were associated with mental health disorders, problem drinking, and trouble sleeping. Most of these observed associations did not differ by specialization. However, the associations between some combat exposures with trouble sleeping differed by occupational specialization and were most consistent among GPF infantrymen and SF personnel.

Combat severity was consistently associated with higher odds of mental health disorders and problem drinking among all occupational specializations. Similar associations were observed in previous studies of military personnel (e.g., [10, 11]). So, while this study replicated the finding that SF personnel [35, 36] and RQ infantrymen [34] were less likely to experience adverse health outcomes, soldiers who experienced high levels of combat had elevated likelihoods of mental health disorders and problem drinking, regardless of occupational specialization. That is, regardless of selection process, level of training, or healthy habits [34], reporting a higher number of individual combat experiences is a substantial risk factor for poor service member behavioral health. Combat severity was also associated with trouble sleeping; however, this association was only found among GPF infantrymen and SF personnel. Perhaps surprisingly, our results indicated that combat severity had less impact on RQ infantrymen’s sleep. Additional studies on combat exposures and sleep are needed, especially given the known bidirectional relationship between sleep and mental health outcomes [50]. In this study, trouble sleeping may be an indicator of larger sleep issues, such as sleep apnea and insomnia. Taken together, these findings highlight the overall negative impact of experiencing numerous types of combat while deployed, for all types of soldiers examined.

Consistent with previous studies, types of combat events (fighting, killing, threat to oneself, death/injury of others) were associated with mental health disorders and problem drinking (e.g., [10, 18, 48]. In addition, the present study examined the relationship between these types of combat events and trouble sleeping. All four types of combat events examined were significantly associated with at least one of these three behavioral health outcomes, independent of the other types of combat events. Most of these associations did not differ by occupational specialization, indicating experiencing these combat events was associated with poorer behavioral health irrespective of self-selection and completion of specialized trainings and missions. However, the association between threat to oneself and trouble sleeping did differ by occupational specialization. Specifically, GPF infantrymen who experienced threat to oneself were more likely to have trouble sleeping compared with GPF infantrymen who did not experience a threat. This finding suggests RQ infantrymen’s and SF personnel’s sleep may be less affected by feeling threatened in combat, possibly due to innate factors or training. In addition, SF personnel have access to advanced training in mental and physical performance enhancement and sleep hygiene, as well as tailored psychological support. Of the four types of combat events examined in this study, killing (combatants and/or noncombatants) had the most consistent relationship with adverse health outcomes, which aligns with previous research [25, 28, 51]. Moreover, these associations did not differ by occupational specialization, indicating that the overall association of killing with behavioral health is similar, regardless of occupational specialization. Such a pattern suggests that specialized training, healthy behaviors, and innate qualities characteristic of specially selected and trained personnel do not protect them from the behavioral health impact of killing.

When we distinguished between types of killing, we found those who were responsible for the death of a noncombatant were more likely to experience mental health disorders, trouble sleeping, and problem drinking compared with those who were responsible for the death of an enemy combatant, after adjusting for fighting, threat to oneself, and death/injury of others. However, only GPF infantrymen and SF personnel who were responsible for the death of a noncombatant had higher likelihoods of trouble sleeping, whereas RQ infantrymen did not. Taken together, these findings support previous research suggesting that killing a noncombatant is particularly associated with poorer health outcomes compared with killing an enemy combatant [10, 25, 27, 28]. Being directly responsible for the death of a noncombatant may go against the service member’s moral code [17, 29, 52], whereas killing an enemy combatant may eliminate a potential threat, serve as a marker of operational success, and be regarded as consistent with the warrior ethos [29]. Events that challenge the moral and ethical foundation of service members can influence all aspects of their lives, contributing to mental and physical health problems [53, 54]. Thus, killing a noncombatant may have a distinct, negative effect on service member well-being. It is important to note, however, that in the current study most of those who killed a noncombatant also killed an enemy combatant, indicating that results may be driven by the cumulative effect of killing both an enemy combatant and noncombatant.

While results from other studies on killing a noncombatant consistently demonstrated an association with adverse health outcomes, findings for killing an enemy combatant were mixed [10, 25, 28, 29]. In the present study, we found those who killed an enemy combatant were more likely to experience mental health disorders, consistent with results from at least one other study [25]. In addition, GPF infantrymen and SF personnel who were responsible for the death of an enemy combatant were more likely to experience trouble sleeping. In contrast, some studies found killing an enemy combatant was not associated with behavioral health problems [29] and had protective effects for PTSD [10], indicating killing an enemy combatant may not necessarily be detrimental to a service member’s psychological health. Understanding the role of killing in predicting the behavioral health of service members can help inform early intervention, clinical screening, and treatment efforts.

Despite the inherent strengths of this study, including a large representative cohort, adjustment for key covariates, and distinct occupational specializations, this study has several limitations. Self-reported survey data may be susceptible to reporting and recall errors. Furthermore, some individuals may not be comfortable reporting combat experiences that may challenge moral or ethical norms, such as being responsible for the death of a noncombatant; however, self-reported combat exposure has been demonstrated to be reliable [55]. Moreover, while service members in specialized units may be less likely to report mental health symptoms for fear of perceived consequences [56], they may be more likely to report accurately in a confidential study [57, 58]. We were also unable to account for survey items related to unit cohesion and leadership due to these items not being included on the Millennium Cohort Study survey during the study period. Likewise, the trouble sleeping outcome was not based on a standardized scale or instrument, but the ascertainment method is consistent with previous published studies [21]. While the present study assessed a range of combat events, there was no measure of combat intensity, such as the number of times a specific combat event was experienced. In addition, survey data used in this study were collected between 2007 and 2013, and may not reflect the current experiences of service members. Finally, based on the cross-sectional nature of the data, the temporal relationship between the assessed exposures and outcomes could not be investigated.

## Conclusions

The current study evaluated the association of combat exposures with behavioral health outcomes among soldiers in three occupational specializations serving in combat roles: GPF infantrymen, RQ infantrymen, and SF personnel. With few exceptions, the study findings indicated that RQ infantrymen and SF personnel have lower odds of adverse behavioral health outcomes compared with GPF infantrymen. Nevertheless, combat experiences were consistently associated with negative health outcomes for all three occupational specializations. The impact of combat on behavioral health was observed not only in terms of overall combat severity but also specific types of combat events, especially being responsible for the death of a noncombatant. Training that incorporates frank dialogue about handling these kinds of experiences and moral reasoning may enable service members to better prepare for difficult decisions that may arise during combat [53, 54]. Those experiencing moral distress after combat may benefit from interventions that emphasize meaning making as well as acknowledging and accepting the moral conflict they may have encountered [53, 59]. Given that previous studies have documented the importance of social connection, unit cohesion [53, 60, 61], and leadership as mitigating factors [54], units and team leaders can also work together to support service members in the wake of such experiences [62]. These recommendations are relevant to GPF infantrymen, RQ infantrymen, and SF personnel, all of whom receive extensive training for combat with the expectation they may engage in killing combatants as part of their operational role. The pattern of these findings should be examined in other military subspecialties, particularly those that emphasize other skill sets and expectations. As suggested by results from the present study, training and other interventions may need to be tailored to specific occupational groups.

## Supporting information

S1 Text Trouble sleeping and previous health conditions sub-analysis. Sub-analysis investigating association between type of combat exposure with trouble sleeping adjusting for history of sleep apnea and previous mental and physical health status, as well as the other covariates (i.e., occupational specialization, race and ethnicity, marital status, education, age, panel, component, and rank).
(DOCX)
