# Supplementary material for: Combat exposure and behavioral health in U.S. Army Special Forces
Source: PLoS One. 2022 Jun 28;17(6):e0270515. doi: 10.1371/journal.pone.0270515 (PMC9239470; doi:10.1371/journal.pone.0270515)
Supplement: S1 Text — Sub-analysis investigating association between type of combat exposure with trouble sleeping adjusting for history of sleep apnea and previous mental and physical health status, as well as the other covariates (i.e., occupational specialization, race and ethnicity, marital status, education, age, panel, component, and rank). (DOCX) [file pone.0270515.s001.docx]

**S1 Text.** **Trouble sleeping and previous health conditions sub-analysis.** Sub-analysis investigating association between type of combat exposure with trouble sleeping adjusting for history of sleep apnea and previous mental and physical health status, as well as the other covariates (i.e., occupational specialization, race and ethnicity, marital status, education, age, panel, component, and rank).

Due to the complex and bidirectional nature between sleep, mental health, and physical health, a sub-analysis was conducted among those who had a previous Millennium Cohort survey (n=2,393) in order to adjust for history of sleep apnea and previous health status in the trouble sleeping models. Adjusted odds ratios were estimated using multivariable logistic regression to determine the association between each type of combat exposure and trouble sleeping, adjusting for covariates as well as three factors from the previous survey (i.e., history of sleep apnea, and mental component summary [MCS] and physical component summary [PCS] scores). MCS and PCS scores evaluate how someone’s mental and physical health effect their day-to-day functioning [1-3]. Sleep apnea was defined as those who answered affirmatively to being diagnosed with sleep apnea by a doctor or other health professional ever or in the last three years, depending on the panel. MCS and PCS scores were assessed using standardized items and scoring algorithms from the Medical Outcomes Short Form 36-Item Health Survey for Veterans (SF-36V) [4, 5]. MCS and PCS scores were summed to have normative values with a mean of 50 and standard deviation of 10, where higher scores are indicative of better health. Models could not be stratified by occupational specialization due to small sample sizes. Overall, the effect estimates once adjusting for history of sleep apnea and health status were similar to those of the main models (Table A). However, the magnitude of association between each type of combat exposure and trouble sleeping was slightly attenuated and the confidence intervals widened; in two cases (threat to oneself and death/injury of others combat events) the effect estimate was not significant (Table A).

Table A. Adjusted odds ratios for trouble sleeping, adjusting for covariates and history of sleep apnea, and MSC and PCS scores.

|  | Main analysis^a^:  Trouble Sleeping^c^ | Sub-analysis^b^:  Trouble Sleeping^c^ |
| --- | --- | --- |
| Model | AOR (95% CI) | AOR (95% CI) |
| Model 1: Combat severity^d^ | n=6,247 | n=2,464 |
| Low (0–6 items) | 1.00 | 1.00 |
| High (7–12 items) | **2.13 (1.90, 2.38)** | **1.94 (1.60, 2.34)** |
| Model 2: Type of combat event^e^ | n=6,201 | n=2,369 |
| Fighting^f^ |  |  |
| No | 1.00 | 1.00 |
| Yes | 1.10 (0.91, 1.34) | 1.11 (0.83, 1.49) |
| Killing^g^ |  |  |
| No | 1.00 | 1.00 |
| Yes | **1.83 (1.62, 2.07)** | **1.81 (1.46, 2.24)** |
| Threat to oneself^h^ |  |  |
| No | 1.00 | 1.00 |
| Yes | **1.29 (1.13, 1.48)** | 1.23 (0.99, 1.53) |
| Death/injury of others^i^ |  |  |
| No | 1.00 | 1.00 |
| Yes | **1.35 (1.10, 1.64)** | 1.32 (0.98, 1.79) |
| Model 3: Type of killing^j,k^ | n=6,201 | n=2,369 |
| Neither | **0.61 (0.54, 0.69)** | **0.61 (0.49, 0.77)** |
| Enemy combatant only | 1.00 | 1.00 |
| Noncombatant | **1.75 (1.42, 2.16)** | **1.63 (1.13, 2.36)** |

AOR, adjusted odds ratio; CI, confidence interval; IED, improvised explosive device

Significant results are shown in bold.

^a^ Results from the main analysis, please see main text for details.

^b^ The sub-analysis was completed only among those who had a previous Millennium Cohort survey and complete data for the additional covariates (i.e., history of sleep apnea, MCS score, and PCS score).

^c^ Trouble sleeping is based on an endorsement of having trouble falling asleep or staying asleep.

^d^ Model 1 (main analysis and sub-analysis) adjusted for occupational specialization, race and ethnicity, marital status, education, age, panel, component, rank; the sub-analysis also adjusted for history of sleep apnea as well as previous MCS and PCS score.

^e^ Model 2 (main analysis and sub-analysis) adjusted for occupational specialization, race and ethnicity, marital status, education, age, panel, component, rank; the sub-analysis also adjusted for history of sleep apnea, as well as previous MCS and PCS score.

^f^ Fighting includes being attacked or ambushed, receiving small arms fire, or clearing/searching buildings.

^g^ Killing includes being directly responsible for the death of an enemy combatant or a noncombatant.

^h^ Threat to oneself includes having an IED explode near you or being wounded or injured.

^i^ Death/injury of others includes seeing dead bodies, handling human remains, knowing someone injured/killed, seeing Americans injured/killed, or unit member injured/killed).

^j^ Type of killing was categorized as “neither,” “enemy combatant only,” and “noncombatant.” Almost all who reported being responsible for the death of a noncombatant also reported being responsible for the death of an enemy combatant.

^k^ Model 3 (main analysis and sub-analysis) adjusted for occupational specialization, fighting, threat to oneself, death/injury of others, race and ethnicity, marital status, education, age, panel, component, and rank; the sub-analysis also adjusted for a history of sleep apnea as well as previous MCS and PCS score.

# References

1. Kazis LE, Lee A, Spiro A, III, Rogers W, Ren XS, Miller DR, et al. Measurement comparisons of the Medical Outcomes Study and Veterans SF-36 Health Survey. Health Care Financ Rev. 2004;25(4):43-58.

2. Kazis LE, Miller Dr Fau - Clark JA, Clark Ja Fau - Skinner KM, Skinner Km Fau - Lee A, Lee A Fau - Ren XS, Ren Xs Fau - Spiro A, 3rd, et al. Improving the response choices on the veterans SF-36 health survey role functioning scales: results from the Veterans Health Study. Journal of Ambulatory Care Management 2004;27(3):263-80.

3. Kazis LE, Miller Dr Fau - Skinner KM, Skinner Km Fau - Lee A, Lee A Fau - Ren XS, Ren Xs Fau - Clark JA, Clark Ja Fau - Rogers WH, et al. Patient-reported measures of health: The Veterans Health Study. Journal of Ambulatory Care Management 2004;27(1):70-83.

4. Ware J, Ma K, Keller SD. SF-36 Physical and Mental Health Summary Scales: a User's Manual. 1993;8:23-8.

5. Ware JE, Kosinski M. Interpreting SF-36 summary health measures: a response. Qual Life Res. 2001;10(5):405-20.
